# A Chemically Recyclable Crosslinked Polymer Network Enabled by Orthogonal Dynamic Covalent Chemistry

**DOI:** 10.1002/anie.202209100

**Published:** 2022-08-23

**Authors:** Yuanxin Deng, Qi Zhang, Da‐Hui Qu, He Tian, Ben L. Feringa

**Affiliations:** ^1^ Key Laboratory for Advanced Materials and Feringa Nobel Prize Scientist Joint Research Center School of Chemistry and Molecular Engineering East China University of Science and Technology 130 Meilong Road Shanghai 200237 China; ^2^ Stratingh Institute for Chemistry and Zernike Institute for Advanced Materials University of Groningen Nijenborgh 4 9747 AG Groningen The Netherlands

**Keywords:** Chemical Recycling, Dynamic Covalent Chemistry, Poly(Disulfide)s, Supramolecular Materials, Sustainable Polymers

## Abstract

Chemical recycling of synthetic polymers offers a solution for developing sustainable plastics and materials. Here we show that two types of dynamic covalent chemistry can be orthogonalized in a solvent‐free polymer network and thus enable a chemically recyclable crosslinked material. Using a simple acylhydrazine‐based 1,2‐dithiolane as the starting material, the disulfide‐mediated reversible polymerization and acylhydrazone‐based dynamic covalent crosslinking can be combined in a one‐pot solvent‐free reaction, resulting in mechanically robust, tough, and processable crosslinked materials. The dynamic covalent bonds in both backbones and crosslinkers endow the network with depolymerization capability under mild conditions and, importantly, virgin‐quality monomers can be recovered and separated. This proof‐of‐concept study show opportunities to design chemically recyclable materials based on the dynamic chemistry toolbox.

## Graphical abstract

Combining two types of dynamic covalent chemistries within a single network enables a chemically recyclable crosslinked material with excellent mechanical performances. Virgin‐quality monomers can be recovered and reused by efficient depolymerization under mild conditions.

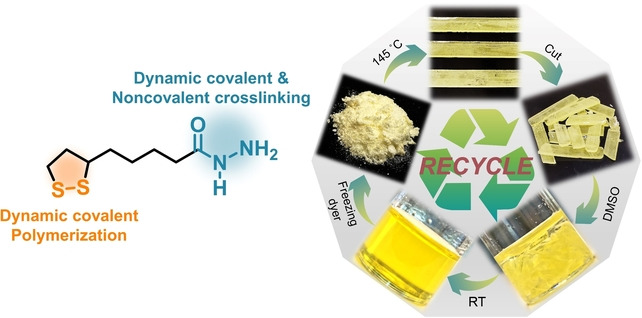

Rising concerns about the health and environmental hazards caused by the accumulation of plastic wastes has confronted us with the challenge to develop sustainable polymers and materials with the intrinsic ability to be readily recycled.^[1]^ Some chemical solutions have been proposed, such as endowing materials with self‐repairing ability to extend the usage cycle of plastics,^[2]^ developing biobased polymers to replace petroleum‐based feedstocks,^[3]^ and enabling chemical recycling in a closed‐loop manner to realize a circular plastic economy.^[4]^ Among these strategies, chemical recycling has been recognized as the ideal option because it enables true sustainability. By molecular engineering the reversible chemical equilibrium between monomers and polymers,[^5^, ^6^] some milestones have been achieved by using catalyst‐assisted or hydrolysis‐induced depolymerization in a few types of polymers such as polyesters,[^4a^, ^4b^] poly(diketoenamine)s,^[4c]^ poly(1,3‐dioxaolane)s,^[4d]^ poly(imine)s,^[4e]^ and poly(disulfide)s.[^4f^, ^4g^] However, recovery of monomers from covalently crosslinked materials is still highly challenging.[^4c^, ^4e^]

Our recent explorations on poly(disulfide)s based on natural thioctic acid (TA) have led to the emergence of a class of intrinsically dynamic materials by taking advantage of disulfide‐mediated reversible polymerizations.^[7]^ A series of versatile supramolecular tools, including hydrogen bonds,[^7a^, ^7e^] ionic bonds,^[7b]^ and metal‐ligand complexation,^[7c]^ has shown their capability of reversible noncovalent crosslinkers for poly(disulfide)s and enabling dynamic functions (e.g. self‐healing ability, responsiveness, and processability). However, the inherent weakness of noncovalent bonds in most cases leads to soft networks but hardly produce robust materials.^[8]^ Introducing covalent crosslinkers can stiffen the network, however with the drawback of lacking reversibility. Although a few pioneering efforts towards the chemical recycling of covalently crosslinked networks by dynamic covalent chemistry have been reported,[^4c^, ^4e^] it is still a formidable challenge to realize robustness with simultaneous decrosslinking and depolymerization of polymer chains, which may provide many opportunities for gels, elastomers, and adhesives.^[9]^ To this end, taking advantage of intrinsically dynamic poly(disulfide)s, our goal is to introduce another dynamic covalent bond as robust and reversible crosslinkers (Figure 1A), thus orthogonalizing two types of dynamic covalent chemistry within a single solvent‐free network.^[10]^

**Figure 1:**
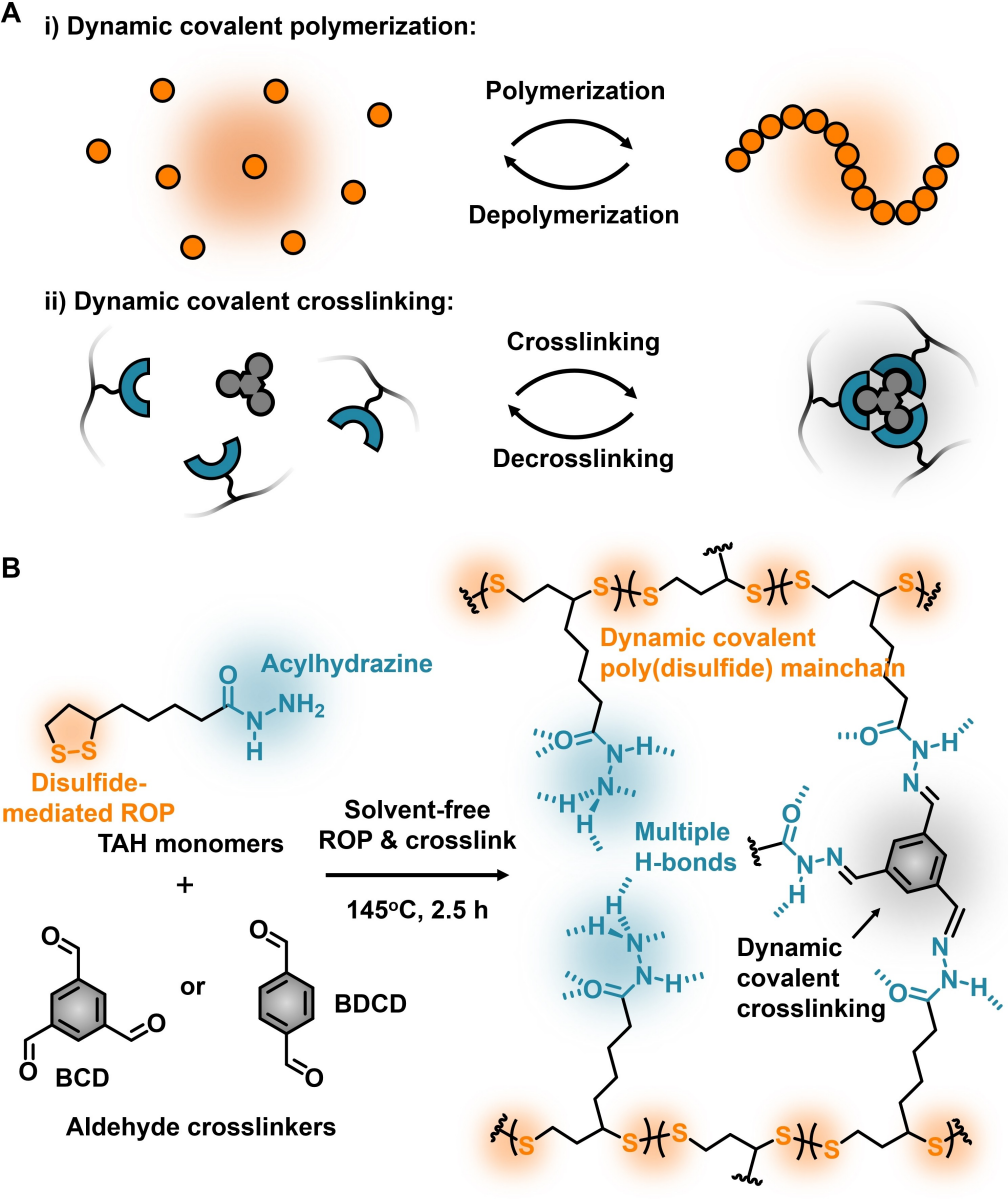
A) Conceptual illustration of dynamic covalent polymerization and crosslinking; B) Molecular structures of the building blocks and the resulting dynamic covalent crosslinked network. The crosslinked network can be prepared by one‐pot solvent‐free melting method and subsequent curing.

Herein, we demonstrate that the acylhydrazone unit, containing a dynamic covalent C=N bond, can act as a robust and reversible crosslinker for poly(disulfide)s (Figure 1B). Introducing a minimal amount (2 % molar ratio) of aldehyde crosslinkers to form acylhydrazones dramatically improves the mechanical performances of poly(disulfide) network. The combination of two types of dynamic covalent chemistry (i.e. mainchain disulfide backbone and sidechain acylhydrazone crosslinkers) jointly enables chemical recyclability to recover virgin‐quality monomers from a covalently crosslinked material. Here the material properties can be tuned via the interplay of reticular hydrogen bonding (due to the acylhydrazones) and two dynamic covalent crosslinking reactions while the ability to recycle is not compromised (Figure 1B). We envision that this strategy of orthogonalizing dynamic chemistry in polymers offers a large chemical space to be explored as well as many opportunities in the design of sustainable plastics and smart materials.

Our very recent study on acylhydrazine‐based TA (TAH) has disclosed the uniqueness of reticular H‐bonds to form high‐modulus noncovalent materials.^[7e]^ However, temperature‐labile H‐bonding crosslinkers leads to visibly softening behaviour upon heating (*T*>40 °C), which significantly limited the applicable temperature window of the materials. To overcome this issue, we envision that, besides forming reticular H‐bonds, acylhydrazine units are also capable of further functionalization with aldehydes to form acylhydrazones, which are also known as dynamic covalent bonds.^[11]^ Therefore, two aldehyde crosslinkers were used, i.e. 1,3,5‐benzenetricarbaldehyde (BCD) and benzene‐1,4‐dicarboxaldehyde (BDCD), because of their commercial availability, rigid backbones, and capability of forming robust acylhydrazones (Figure 1). The preparation of poly(TAH) crosslinked by different amounts of BCD or BDCD (1–10 % molar ratio of TAH monomers) was performed by a one‐pot solvent‐free melting method based on a modification of our previous procedures^[7a]^ (see details in Supporting Information). Upon cooling down to room temperature, yellow translucent solid materials were formed. FT‐IR spectroscopy was used to characterize the bond formation in the resulting solvent‐free network (Figure 2A). With the increasing amounts of BCD from 1 % to 10 % molar ratio, two distinctive IR bands appeared at 1626 cm^−1^ and 1597 cm^−1^, respectively, attributed to the *υ*_C=N_ and *υ*_C=O_ bands of the formed acylhydrazone units.^[12]^ The broad absorption bands of *υ*_N‐H_ at around 3300 cm^−1^ confirmed the abundant existence of H‐bonds in the network, which should be jointly contributed by acylhydrazines and acylhydrazones as revealed by their low‐wavenumber *υ*_C=N_ and *υ*_C=O_ bands. Notably, the absence of vibration band of hydroxyl groups confirmed that there was no free or structural water molecules^[13]^ in the resulting network. X‐ray diffraction (XRD) and synchrotron‐radiation small‐angle X‐ray scattering (SAXS) measurements confirmed the amorphous network without notable microphase separation (Figure S1 and S2). Solubility tests revealed the lack of solubility of the materials in most organic solvents (Figure S3). Thermogravimetry analysis showed the enhanced decomposition temperature after introducing aldehyde crosslinkers (Figure S4). These experimental data jointly confirmed the successful covalent crosslinking of poly(TAH) by forming acylhydrazones in the solvent‐free network.

**Figure 2:**
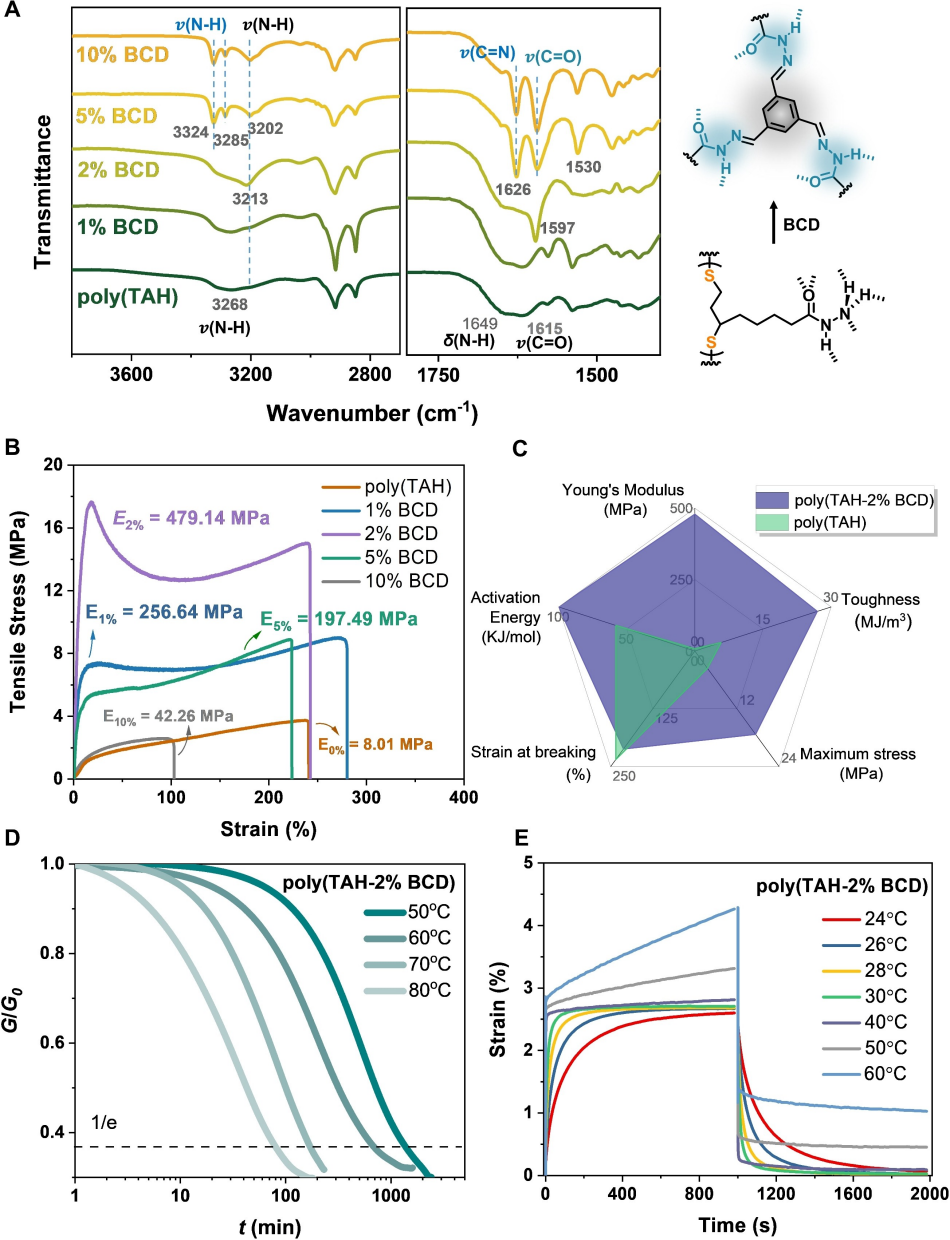
Structural and mechanical characterization of the resulting crosslinked materials. A) FT‐IR spectra of the resulting poly(TAH) network with and without BCD crosslinkers; B) Stress‐strain curves of the resulting poly(TAH) with different amounts of BCD crosslinkers (strain rate=10 mm min^−1^); C) Radar diagram comparison of the mechanical performance of poly(TAH) before and after crosslinking by 2 % molar ratio BCD; D) Temperature‐dependent stress relaxation curves for poly(TAH‐2 %BCD); E) Creep‐recovery plots for poly(TAH‐BCD) at different temperatures under 10 KPa.

Investigating the mechanical properties of the crosslinked materials, tensile stress‐strain curves showed the significantly improved mechanical performances after introducing BCD and BDCD crosslinkers (Figure 2B and Figure S5–S6). Samples with 2 % molar ratio BCD exhibited the optimal performance enhancement (Figure 2C), including 60‐fold in stiffness, 4.7‐fold in toughness, 4.8‐fold in maximum stress and no compromise on the stretchability compared with poly(TAH) without crosslinkers. The mechanism responsible for the improvement of mechanical properties may be due to the synergy of two kinds of dynamic crosslinking interactions, e.g. the reticular H‐bonds of acylhydrazine units and dynamic covalent acylhydrazone linkers. In contrast, a higher ratio of crosslinkers showed a decreased Young's modulus and yield modulus. This side effect may stem from the lower number of acylhydrazine units and reticular H‐bonds^[7e]^ that also contribute to the network crosslinking, showing the delicate interplay of dynamic covalent crosslinking and H‐bonds in the network.

To further understand the dynamic properties of the crosslinked network, rheological experiments were performed. The Poly(TAH‐2 %BCD) network exhibited a typical rubbery plateau in the temperature region from 25 °C to 140 °C (Figure S7). To quantify the dynamic behaviours of the chain mobility, the averaged apparent activation energy (*E*_a_) for chain mobility was measured through a series of temperature‐variable relaxation experiments on dynamic mechanical analysis (DMA, Figure 2D). Fitting with the Arrhenius's equation, the *E*_a_ of the poly(TAH‐2 %BCD) was evaluated as 100 kJ mol^−1^ (Figure S8), which was higher than that of poly(TAH) without BCD crosslinkers (50 kJ mol^−1^),^[7e]^ indicating that the presence of acylhydrazone moieties enhanced interchain crosslinking and meanwhile preserved chain mobility (i.e. dynamic functions). Temperature‐dependent creep‐recovery (Figure 2E) experiments showed the notably improved anti‐creep and strain‐recovery ability after introducing BCD crosslinkers (Figure 2E and Figure S9), thus broadening the temperature window and application scope of the materials. To illustrate the reprocessing ability, polymer fragments were “repaired” by hot‐melting under 145 °C (Figure S10A), resulting in virgin‐quality materials as revealed by the completely overlapped curves in DMA (Figure S10B). Further tensile experiments also confirmed the good reprocessing ability of the materials (Figure S10C). These results indicated that the resulting covalently crosslinked network can be dynamically adaptable, and exhibit mechanical reprocessability and repairability at higher temperatures.

It has been recognized that a more ideal way to recycle polymers is chemical depolymerization and monomer recovery because it leads to closed‐loop sustainability.^[14]^ When studying poly(TAH) polymers by ^1^H NMR, we surprisingly discovered their self‐depolymerization ability in polar solvents (e.g. dimethylformamide (DMF) and dimethyl sulfoxide (DMSO)), as revealed by the ^1^H NMR spectra of polymer solutions showing no polymeric species but monomers (Figure 3A). This intrigued us since there was no external catalysts added in the system and the scission of linear disulfide bonds (around 60 kcal mol^−1^)^[15]^ undoubtedly requires the acceleration by catalysis at room temperature. Considering the moderate nucleophilicity of acylhydrazine units, we inferred that the sidechain acylhydrazine units may be responsible for the auto‐depolymerization mechanism (Figure 3B). To verify this hypothesis, an acidified DMSO solution (pH 2–3) was used to protonate acylhydrazine units and thus to reduce the nucleophilicity (Figure S11). As a result, poly(TAH) samples showed swelling ability instead of dissolution and no monomer formation, indicating the protonation‐inhibited depolymerization and in accordance with the proposed mechanism of acylhydrazine‐catalysed depolymerization (Figure 3B).

**Figure 3:**
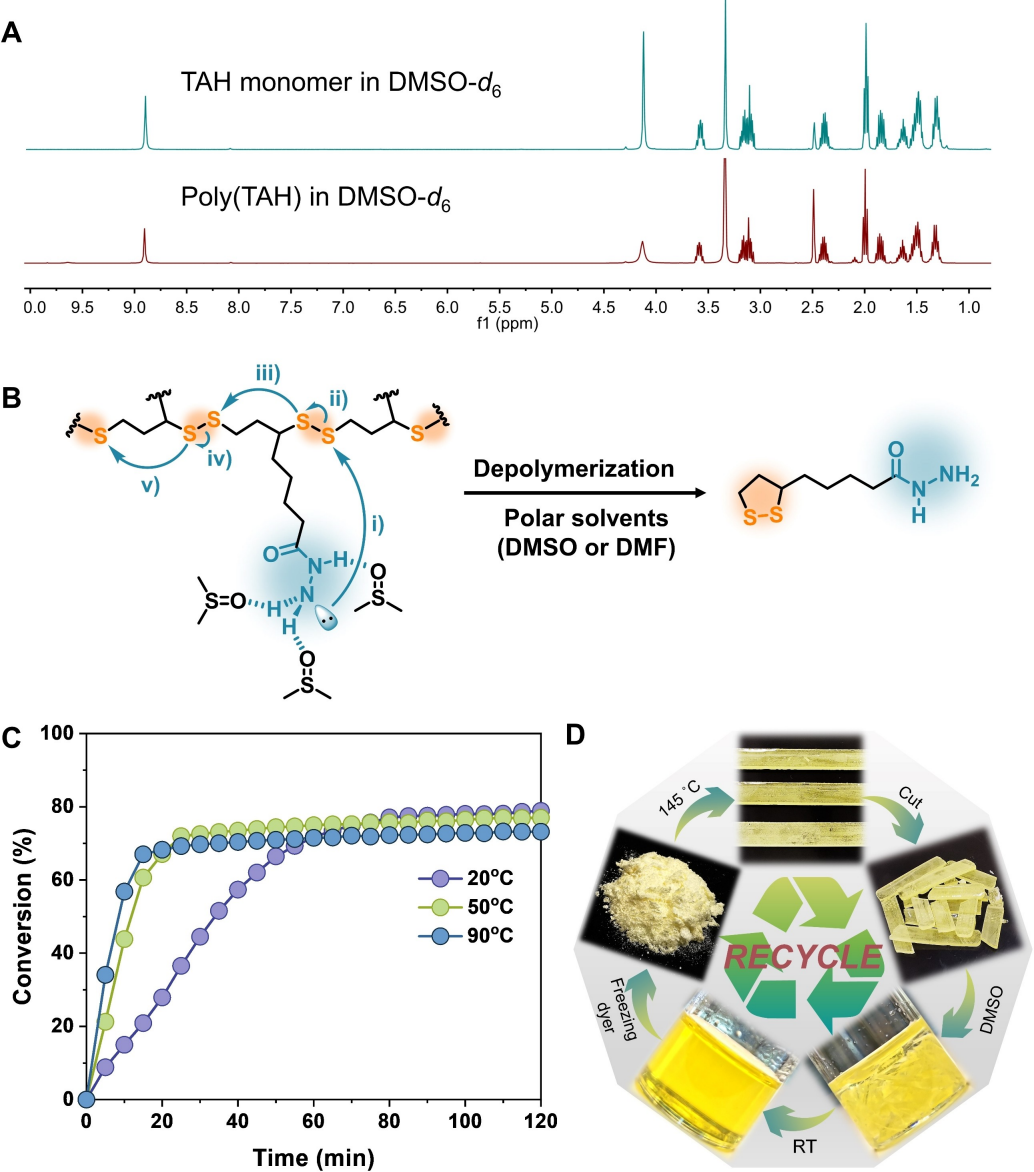
Depolymerization ability of poly(TAH). A) ^1^H NMR spectra of the TAH monomers and poly(TAH) solution in *d*_6_‐DMSO, indicating the quantitative depolymerization of poly(TAH) polymers; B) Proposed depolymerization mechanism of the poly(TAH) polymers in polar solvents; C) Temperature‐dependent depolymerization kinetic curves of poly(TAH) in DMSO detected by UV/Vis spectroscopy; D) Graphic scheme showing the closed‐loop chemical recycling of poly(TAH) homopolymers.

Taking advantages of the distinctive absorption band of TAH monomers at 330 nm,^[16]^ the depolymerization process can be easily detected by UV/Vis spectroscopy (Figure 3C and S12). Temperature‐dependent depolymerization experiments showed that the depolymerization process is completed in approximately one hour at 20 °C and the period can be shortened in 20 min by heating the material to 50 °C. Using freeze‐drying method, monomer powders are recovered from the depolymerization mixtures in DMSO solution with a high yield (78 %, Figure 3D). A series of spectroscopic characterizations, including ^1^H NMR, FT‐IR, and DSC, jointly confirmed the virgin quality of the recovered TAH monomers (Figure S13–S15).

Furthermore, we investigated the chemical recycling of more robust poly(TAH‐2 %BCD) network featuring a tris‐aldehyde cross‐linker (Figure 1B). The presence of BCD crosslinkers caused partial decrease of recycling yield (49 %) as the acylhydrazone crosslinkers were showing robustness in DMSO solutions (Figure S16). Therefore, to enable the efficient dynamic covalent decrosslinking, external nucleophiles (i.e. hydroxylamine and hydrazine) were used to decrosslink the network by competing exchange (Figure 4A).^[17]^ Model reaction detection based on ^1^H NMR spectroscopy (Figure S17, S18) showed nearly quantitative conversion of acylhydrazones into oximes in the case of hydroxylamine and also high conversion (87 %) in the case of hydrazine. Furthermore, polymer recycling experiments have been performed by addition of hydrazine and catalytic amount of TFA in DMSO, offering a versatile and mild recycling method with an enhanced monomer isolation yield of 66 % (Figure 4B). Notably, the isolated monomers exhibited high quality and purity as indicated by the fully consistent spectroscopic properties including FT‐IR (Figure 4C) as well as high crystallinity in XRD (Figure S19) and consistent melting peak in DSC curve compared with original monomers (Figure 4D). The recycled virgin quality monomers can be used as the starting materials for polymer preparation, affording materials with similar mechanical properties (Figure S20). These experimental data unambiguously confirm the robustness of our design strategy for constructing orthogonal dynamic covalent networks^[18]^ with easy chemical recyclability.

**Figure 4:**
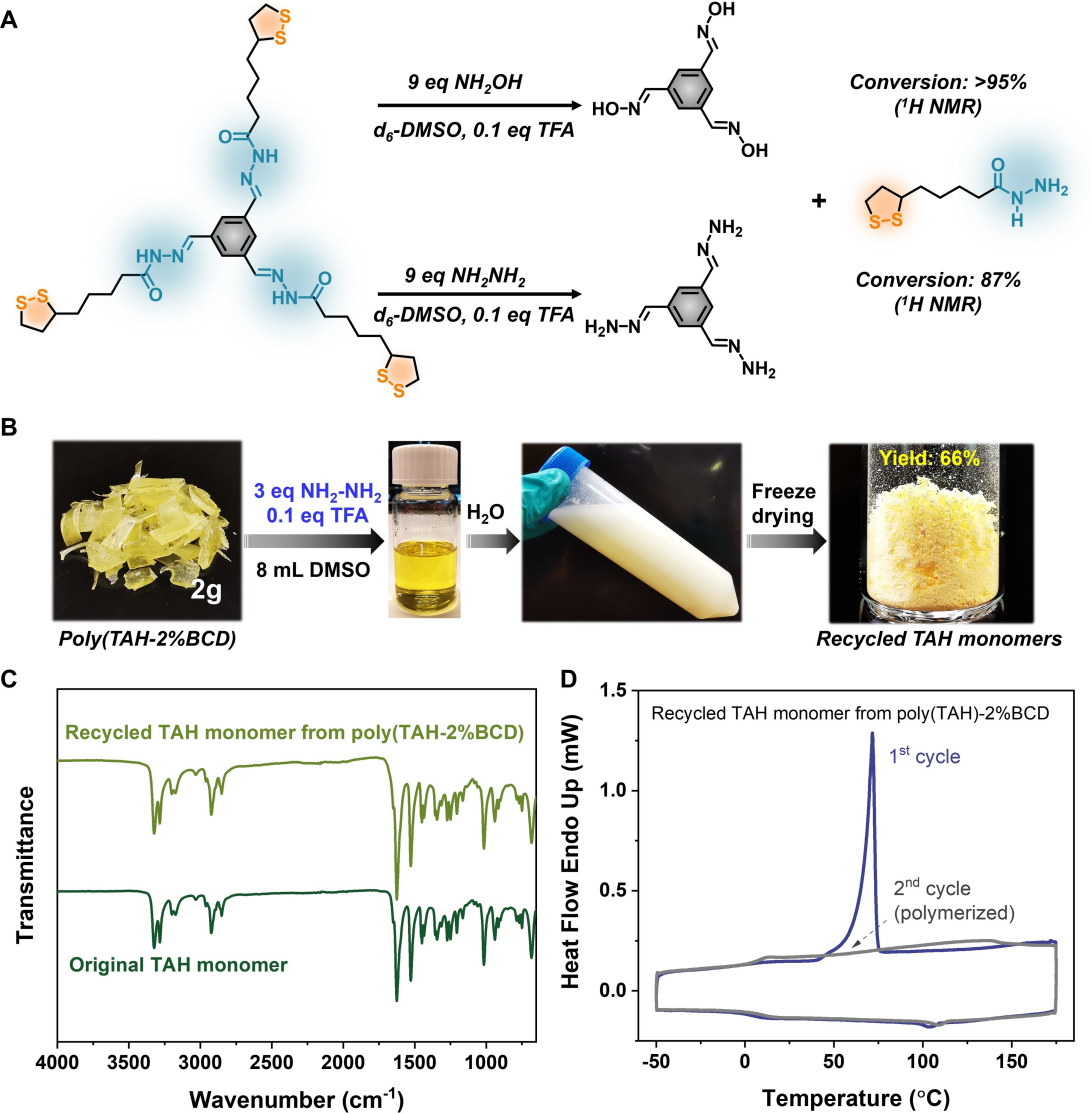
Chemical recycling of crosslinked network. A) Small‐molecule model reactions showing the efficient dynamic covalent exchange based on hydroxylamine and hydrazine as the competing units; B) Photographs of the chemical recycling of poly(TAH‐2 % BCD) with an isolation yield of 66 %; C) FT‐IR spectra of the original and recovered TAH monomers suggesting the high purity of the recycled monomers; D) DSC curve of the recycled TAH monomers. The sharp melting peak at the 1^st^ cycle indicated the virgin quality of the recycled TAH monomers.

In summary, we designed orthogonal dynamic covalent polymeric materials by integrating disulfide‐mediated reversible polymerization with acylhydrazone‐based dissociable crosslinking. The mechanical properties of the network can be readily tuned by varying the amount of acylhydrazones with a dual function acting as supramolecular multiple H‐bonding units and dynamic covalent crosslinkers, enabling a crosslinked network with high mechanical robustness, toughness, and adaptability. Due to the orthogonal existence of two types of dynamic covalent bonds, the covalently crosslinked network can be dissociated and depolymerized into monomers under mild conditions including highly robust ones using small molecule external nucleophiles. Virgin‐quality monomers can be separated from the solutions after degradation in good yields. It should be mentioned that this dynamic material still exhibits creeping and flowing properties at high temperatures, even if the temperature window has been expanded by introducing covalent acylhydrazone crosslinkers. The inherent conflicts between thermal stability and dynamic properties provides still a challenge towards industrially applicable materials. We envision that this proof‐of‐concept study may offer a distinctive approach to the development of high‐performance chemically recyclable plastics.

## Conflict of interest

The authors declare no conflict of interest.

## Supporting information

As a service to our authors and readers, this journal provides supporting information supplied by the authors. Such materials are peer reviewed and may be re‐organized for online delivery, but are not copy‐edited or typeset. Technical support issues arising from supporting information (other than missing files) should be addressed to the authors.

Supporting Information

## Data Availability

The data that support the findings of this study are available from the corresponding author upon reasonable request.
