# Supplementary material for: A Chemically Recyclable Crosslinked Polymer Network Enabled by Orthogonal Dynamic Covalent Chemistry
Source: Angew Chem Int Ed Engl. 2022 Aug 23;61(39):e202209100. doi: 10.1002/anie.202209100 (PMC9804754; doi:10.1002/anie.202209100)
Supplement: Supplementary file 1 — Supporting Information [file ANIE-61-0-s001.pdf]

Angewandte Chemie
Eine Zeitschrift der Gesellschaft Deutscher Chemiker

Supporting Information

A Chemically Recyclable Crosslinked Polymer Network Enabled by Orthogonal Dynamic Covalent Chemistry

Y. Deng, Q. Zhang, D.-H. Qu, H. Tian, B. L. Feringa**

CONTENT for Supporting Information:

1. **Materials**
2. **Instruments and methods**
3. **Synthetic procedures**
4. **Methods for chemical recycling**
5. **Supporting figures**

1. Materials

The key feedstock (±) -α-thioctic acid (TA) was used as received from Aladdin® with a Reagent Grade (99%). N,N'-disuccinimidyl carbonate, triethylamine (>99%) (NHS), hydroxylamine solution (50wt.% in H_2O), hydrazine monohydrate (79%), 1,3,5-benzenetricarbaldehyde (BCD) and benzene-1,4-dicarboxaldehyde (BDCD) were obtained from commercial sources (Sigma-Aldrich and TCI) and used as received without further purification. Solvents used for reactions and spectroscopy were HPLC grade. Solvents for NMR spectroscopy were used as received from Sigma-Aldrich.

2. Instruments and Methods

Attenuated Total Reflection (ATR) measurements were performed on a Perkin-Elmer FT-IR Spectrometer 400. Solid polymer samples were added onto the surface of sample platform and the background of the sample platform was corrected. The measurements were performed at room temperature (25 °C).

Differential Scanning Calorimetry (DSC) were carried out on a TA instruments DSC Q1000 in a dry nitrogen atmosphere. Samples were cycled from -50 °C to 175 °C at a rate of 5 °C/min. The glass transition temperature was determined by taking the midpoint of the reversible endotherm of the 2nd heating scan for each sample.

Thermogravimetric Analysis (TGA) was carried out on a TGA5500 (TA Instruments, USA) in a nitrogen atmosphere with a temperature range from 20 °C to 600 °C with a heating rate of 10 °C/min.

X-ray Diffraction (XRD) Experiments were performed using a rotating anode X-ray powder diffractometer (18KW/D/max2550VB/PC) equipped with a copper target 18KW (450mA), a fully automated curved (plate) crystal graphite monochoromator and a programmed variable slit system.

Dynamic Mechanical Analysis (DMA) was performed on a PerkinElmer DMA Q8000 operating in tension mode. Temperature ramps were carried out at a constant rate of 3 °C/min from -100 °C to 100 °C for all samples with a displacement of 2 μm, a fixed frequency of 1 Hz, and a force multiplier of 1.5. Stress relaxation analysis tests were performed between 50 °C and 80 °C with a constant strain of 1%.

Rheological Tests were performed on a TA Instruments Discovery HR-2 rheometer. Polymer samples were placed under a 20-mm-diameter parallel plate with a gap of 0.1-2.0 mm. Temperature dispersion tests were carried out in a range from -20 °C to 160 °C (5 °C/min) at a constant frequency of 1 Hz with an applied strain of 0.1%. Frequency sweep tests were conducted in a range from 626 rad/s to 6.28×10^3 rad/s at room temperature with an applied strain of 0.1%. Creep recovery tests were carried out between 24 °C and 60 °C with a constant 10 kPa force. The creep test ranges from 0 s to 990 s and the recovery test ranges from 1000 s to 1180 s for the poly(TAH-BCD) polymers. The creep test ranges from 0 to 390 s and the recovery test ranges from 400 s to 1180 s for the poly(TAH) polymers.

Small Angle X-Ray Scattering (SAXS) were performed on the BL19U2 SAXS beamline at Shanghai Synchrotron Radiation Facility (Shanghai, China).

Solution-Phase Nuclear Magnetic Resonance (NMR) ^{1}H NMR and ^{13}C NMR spectra were measured on a Bruker AV-400 (^{1}H: 400 MHz, ^{13}C: 100 MHz) spectrometer at room temperature. Chemical shift values (δ) are reported in parts per million (ppm) with the solvent resonance as the internal standard (DMSO: δ 2.49 for ^{1}H, δ 39.52 for ^{13}C). The following abbreviations (and their corresponding combination) are used to indicate signal multiplicity: s (singlet), d (doublet), t (triplet), q (quartet), m (multiplet), and br (broad).

Mechanical Tensile Tests were carried out on an Instron 4301 tensile machine mounted with a maximum 5 KN detection cell. The data was recorded in real time by a wire-connected computer system. Unless otherwise noted, samples were tested at a fixed tensile speed of 10 mm/min. Tensile bars (60 mm × 5 mm × 2 mm) were prepared using a Teflon mold with multiple cells to enable parallel conditions. The tensile measurement was carried out at ambient condition for all samples and each measurement was repeated with at least three independent samples.

3. Synthetic Procedures

Preparation of poly(TAH-BCD) and poly(TAH-BDCD): In a typical procedure, 5 g (0.023 mol) TAH monomer[1] and various molar ratio of aldehyde crosslinkers (1%, 2%, 5%, and 10%) were added into a 15 mL Teflon vial. The mixture was then heated by a metal heating block with a constant temperature of 145 °C for 2.5 h to obtain homogeneity and the resulting liquid was quickly transferred into a Teflon mold and cooled to room temperature to form free-standing polymer samples.

Preparation of Model Molecule:

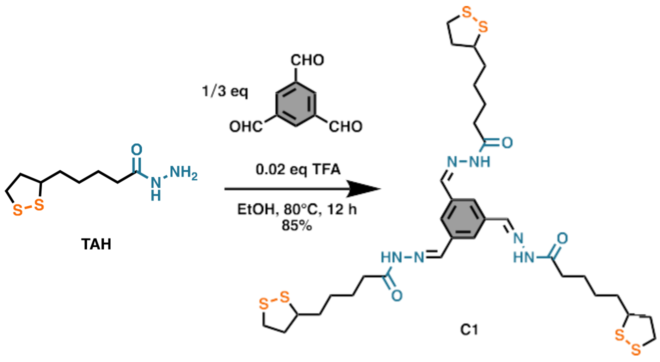

Preparation of compound **C1**: 5-(1,2-dithiolan-3-yl)pentanehydrazide (TAH) (1.0 eq, 2.0 g, 9.0 mmol), 1,3,5-benzenetricarbaldehyde (BCD) (1/3 eq, 0.5 g, 3.0 mmol) and trifluoroacetic acid (0.02 eq, 15 L) were dissolved in EtOH (100 mL). The mixture was heated at reflux for 12 h. After cooling, the resulting solid was filtered and washed with EtOH to yield light yellow solid compound **C1** (2.0 g, yield = 85%). Major isomer: ^{1}H NMR (400 MHz, DMSO-d_6) δ 11.36 (d, 1H), 7.91 (d, 1H), 8.03 (m,1H), 3.6 (m, 1H), 3.12 (m, 2H), 2.65 (t, 1H), 2.4 (m, 1H), 2.22 (m, 1H), 1.86 (m, 1H), 1.70 (m, 1H), 1.59 (m, 3H), 1.41 (m, 2H). Minor isomer: ^{1}H NMR (400 MHz, DMSO-d_6) δ 11.47 (t, 1H), 8.20 (d,1H), 7.96 (m, 1H), 3.6 (m, 1H), 3.12 (m, 2H), 2.65 (t, 1H), 2.4 (m, 1H), 2.22 (m, 1H), 1.86 (m, 1H), 1.70 (m, 1H), 1.59 (m, 3H), 1.41 (m, 2H). Major isomer: ^{13}C NMR (151 MHz, DMSO-d_6) δ 169.11, 141.90, 135.88, 126.45, 56.62, 40.53, 38.58, 34.62, 32.16, 28.79, 25.22. Minor isomer: ^{13}C NMR (151 MHz, DMSO-d_6) δ 174.73,

145.09, 135.88, 126.45, 56.57, 40.53, 38.58, 34.62, 32.16, 28.95, 24.53. HRMS (APCI) (m/z): $[M + H]^+$ calcd for $C_{33}H_{48}N_6O_3S_6$ + H: 769.21849, found: 769.21928.

4. Methods for Chemical Recycling

Chemical Recycling Process of poly(TAH): poly(TAH) specimens (2.0 g) were cut into pieces and then were immersed in DMSO solvent (5.0 mL). The mixture was stirred at room temperature for 4 h to result a transparent yellow solution. The solution was diluted with 30 mL H_2O to produce a yellow precipitate. The compound was dried by freeze drying to yield a yellow solid monomer (1.6 g, 78%). Characterization data identical with starting monomer.

Chemical Recycling Process of poly(TAH-2% BCD): poly(TAH-2% BCD) specimens (2.0 g) were cut into pieces and then immersed in DMSO solvent (5.0 mL). The mixture was stirred at 50 °C for 12 h to give a yellow solution. The solution was diluted with 30 mL H_2O to produce a yellow precipitate. The crude product was filtered and then was dried by freeze drying to yield a yellow solid monomer (0.98 g, 49%).

Chemical Recycling Process of poly(TAH-2% BCD) with External Nucleophlies: poly(TAH-2% BCD) specimens (2.0 g) were cut into pieces and then immersed in DMSO solvent (8.0 mL). Hydrazine (3.0 eq, 1.6 mL) and trifluoroacetic acid (0.1 eq, 69 μL) were added into the mixture. The mixture was stirred under 50 °C for 5 h to result a transparent yellow solution. The solution was diluted with 40 mL H_2O to produce a yellow precipitate. The compound was filtered and dried by freeze drying to yield a yellow solid monomer (1.3 g, 66%).

5. Ethical approval

There is no animal/human experiment involved in this research.

6. Supporting Figures

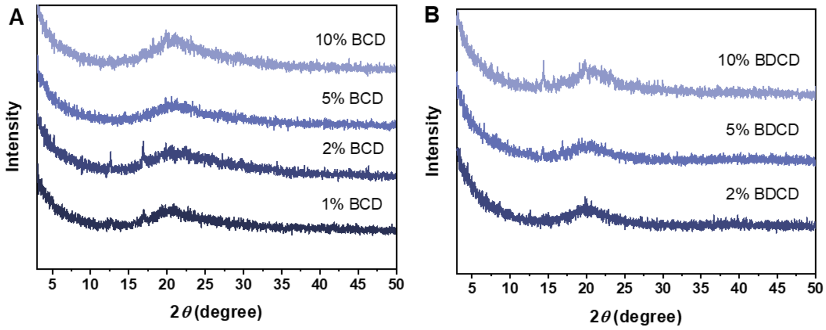

Figure S1. XRD analysis of poly(TAH-BCD) and poly(TAH-BDCD) with various molar ratio, revealing the absence of crystallinity, indicating the polymer network is amorphous.

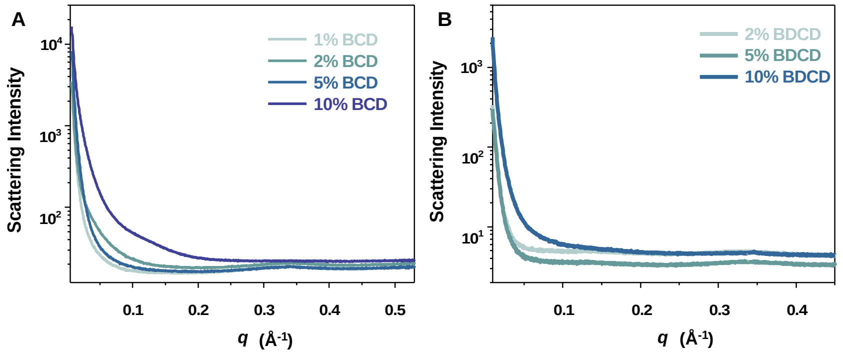

Figure S2. SAXS analysis of poly(TAH-BCD) and poly(TAH-BDCD) with various molar ratio, revealing the amorphous network without notable microphase separation.

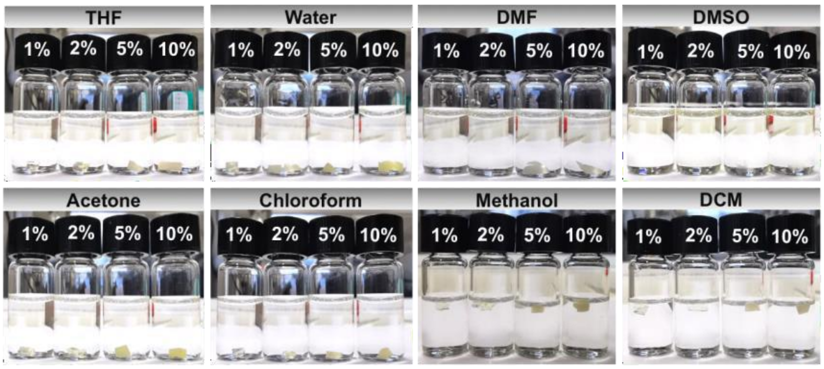

Sample	THF	Water	DMF	DMSO	Acetone	Chloroform	Methanol	DCM
PolyTAH-1% BCD	x	x	O	O	x	x	x	x
PolyTAH-2% BCD	x	x	O	O	x	x	x	x
PolyTAH-5% BCD	Δ	Δ	Δ	partial	Δ	Δ	Δ	Δ
PolyTAH-10% BCD	Δ	Δ	Δ	partial	Δ	Δ	Δ	Δ

Figure S3. Solubility test of the resulting poly(TAH-BCD) with various amount of crosslinkers. The polymer samples soaked in different solvents for 24 h (“O” means totally soluble; “Δ” means swelling; “×” means insoluble).

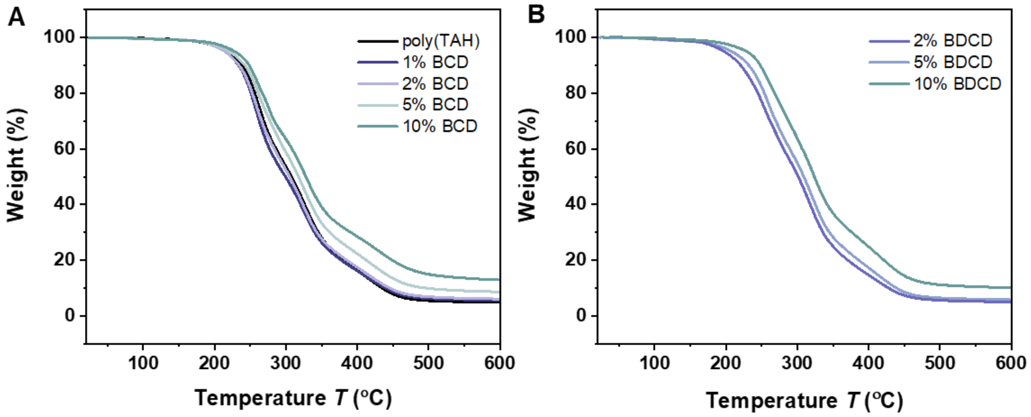

Figure S4. TGA analysis poly(TAH) with various molar ratio of BCD and BDCD.

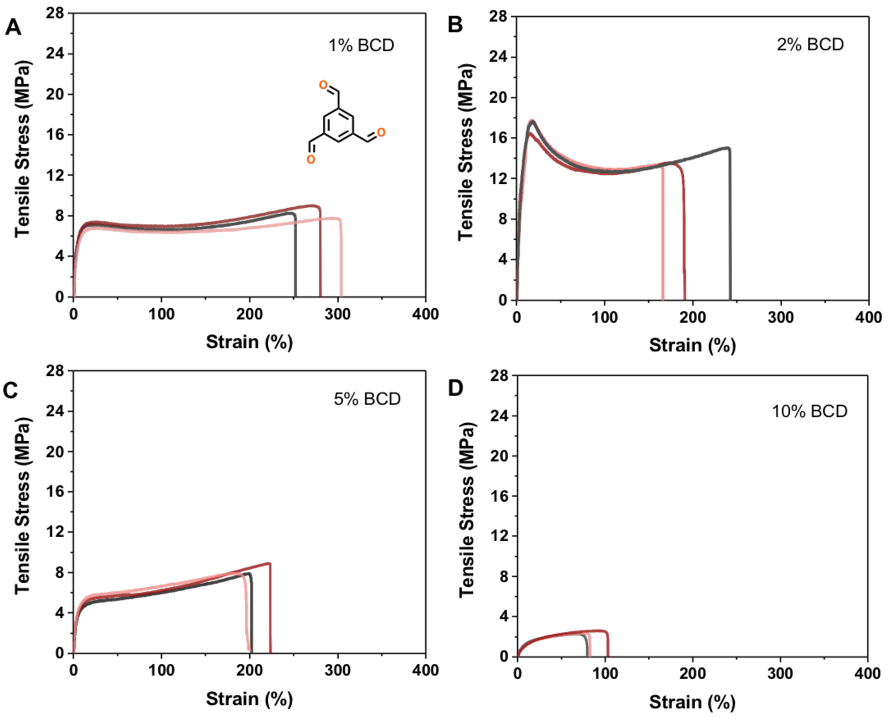

E

sample	tensile strength (σ_{max}, Mpa)	tensile strain (ε_b, %)	young's modulus (E, MPa)	tensile toughness (U_T, MJ·m^{-3})
PolyTAH-1% BCD	8.34 ± 0.51	278.69 ± 21.19	256.64 ± 4.27	19.68 ± 1.46
PolyTAH-2% BCD	17.30 ± 0.58	211.90 ± 23.63	479.14 ± 5.55	26.95 ± 4.54
PolyTAH-5% BCD	8.24 ± 0.45	208.73 ± 10.55	197.49 ± 1.63	13.32 ± 1.07
PolyTAH-10% BCD	2.39 ± 0.14	88.47 ± 10.37	42.26 ± 1.58	1.71 ± 2.99

Figure S5. (A-D) Stress-strain curves of poly(TAH) with various ratio of BCD; (E) A summary of the mechanical data of the poly(TAH-BCD).

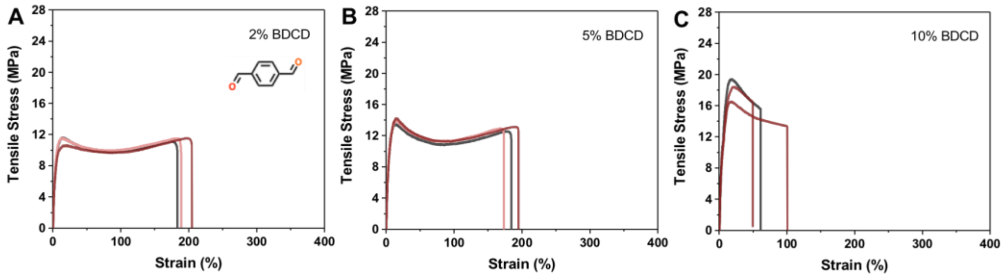

D

sample	tensile strength (σ_{max}, Mpa)	tensile strain (ε_b, %)	young's modulus (E, MPa)	tensile toughness (U_T, MJ·m^{-3})
**PolyTAH-2% BDCD**	11.38 ± 0.19	192.53 ± 8.86	353.93 ± 10.48	17.96 ± 0.80
**PolyTAH-5% BDCD**	12.87 ± 0.24	184.20 ± 8.78	392.06 ± 8.15	21.78 ± 1.64
**PolyTAH-10% BDCD**	18.13 ± 1.19	70.59 ± 21.84	426.76 ± 8.77	10.68 ± 2.64

Figure S6. (A-C) Stress-strain curves of poly(TAH) with various ratio of BDCD; (D) A summary of the mechanical data of poly(TAH-BDCD). The different crosslinking topologies of BDCD (dialdehydes) and BCD (trialdehydes), i.e. bis-functionalized versus tris-fucntionalized crosslinker, should be responsible for the different mechanical performances of the resulting materials.

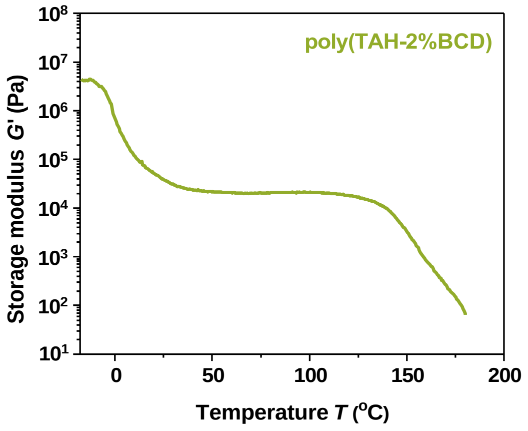

Figure S7. Temperature-dependence of rheological curves of G' at 1 Hz.

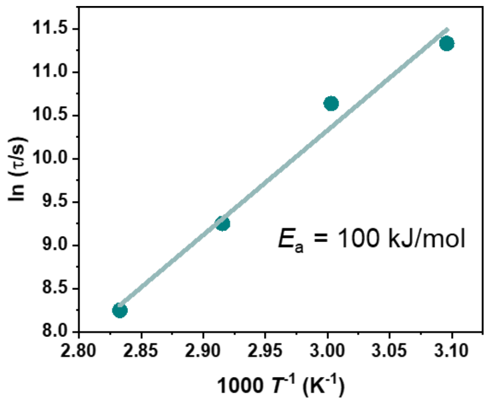

Figure S8. Arrhenius-type behavior of Poly(TAH-2%BCD) with apparent activation energy of 100 kJ/mol.

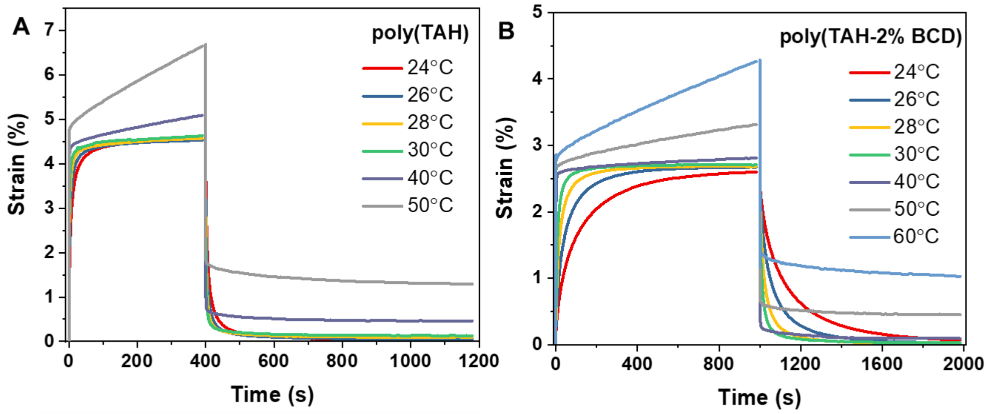

Temperature	Viscosity (Pa s)
50 °C	2.3 x 10^6
40 °C	6.5 x 10^6
30 °C	2.2 x 10^7

Temperature	Viscosity (Pa s)
60 °C	7.8 x 10^6
50 °C	1.9 x 10^7
40 °C	6.8 x 10^7

Figure S9. Creep-recovery plots and viscosities at different temperature for (A) poly(TAH) and (B) poly(TAH-2% BCD) (σ = 10 KPa, viscosities at different temperature were extracted from fits on the linear regimes of the plots[2]).

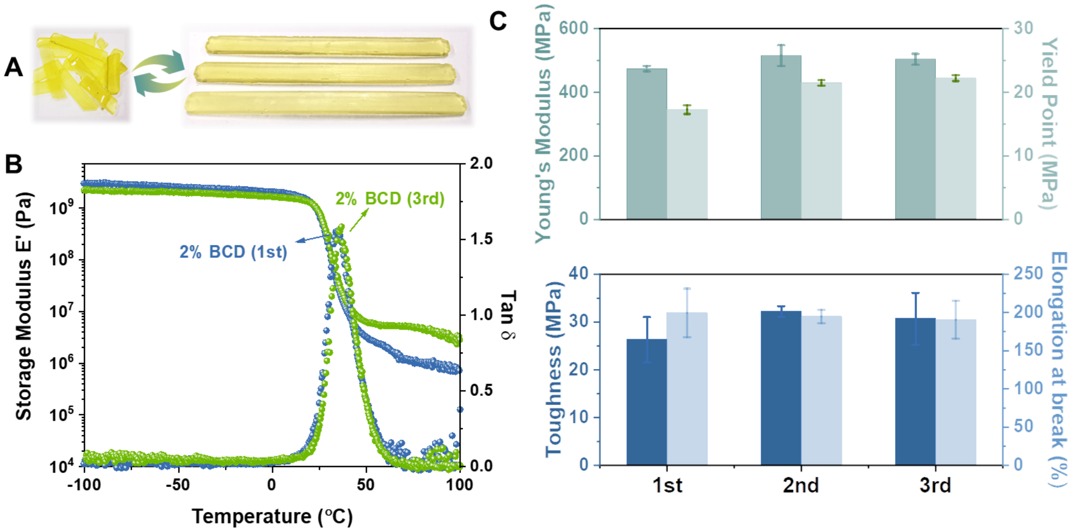

Figure S10. (A) Mechanical recycling process of poly(TAH-2% BCD) by hot-melting under 145 °C; (B) DMA data of poly(TAH-2% BCD) before and after mechanical recycling; (C) Mechanical performance of the mechanical recyclable poly(TAH-2% BCD) after repeating multiple cycles.

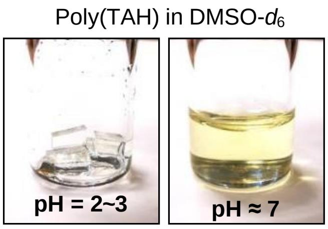

Figure S11. Photographs of poly(TAH) in DMSO with distinct pH value. Poly(TAH) swelled in acidic DMSO (pH = 2-3) (the solvent was removed for clear presentation), while fully dissolved at neutral pH (pH = 7).

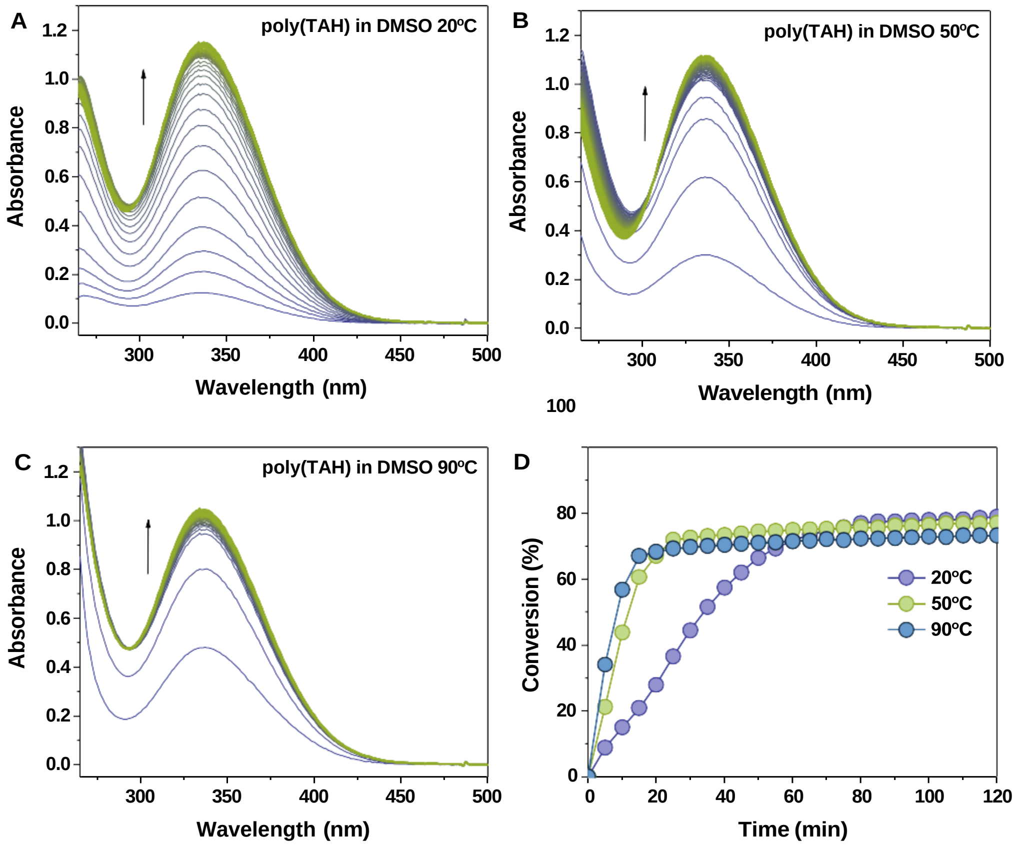

Figure S12. (A-C) Real-time detected UV-vis spectra of the DMSO solution of the degraded poly(TAH) at different temperatures; (D) Kinetic curves of the degraded poly(TAH) in DMSO solution at different temperatures.

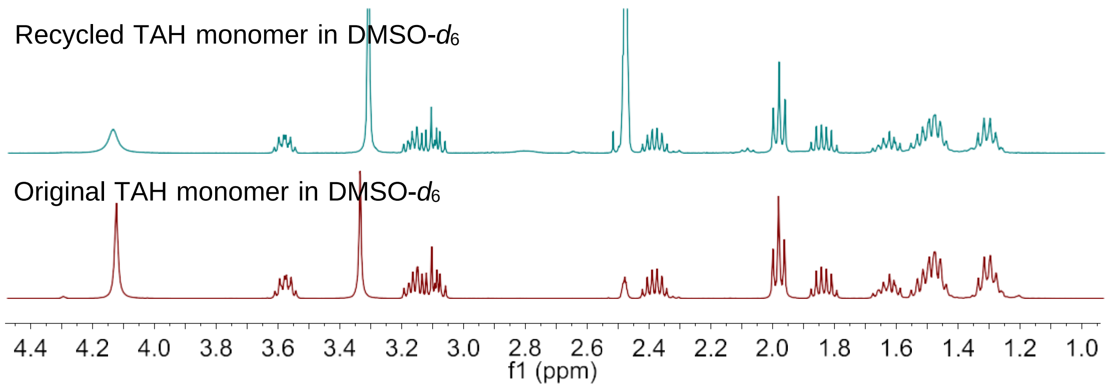

Figure S13. ^{1}H NMR spectra of the original and chemical recycled TAH monomer from poly(TAH) (DMSO-d_6, 400 MHz, 298 K).

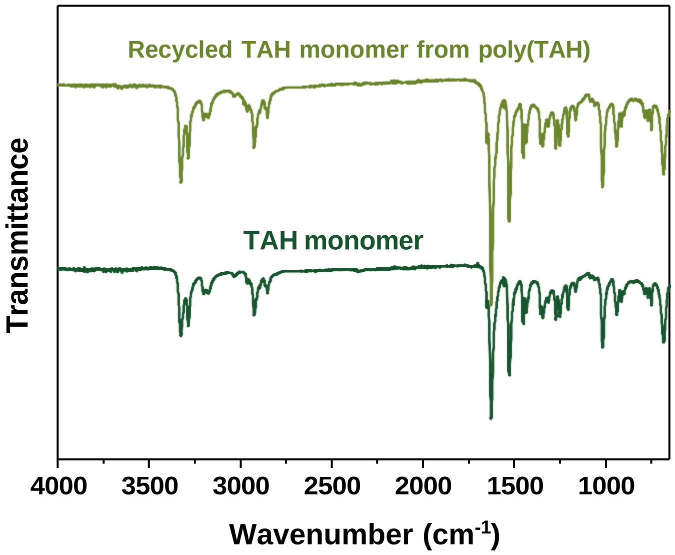

Figure S14. ATR data of the original and chemical recycled TAH monomer from poly(TAH).

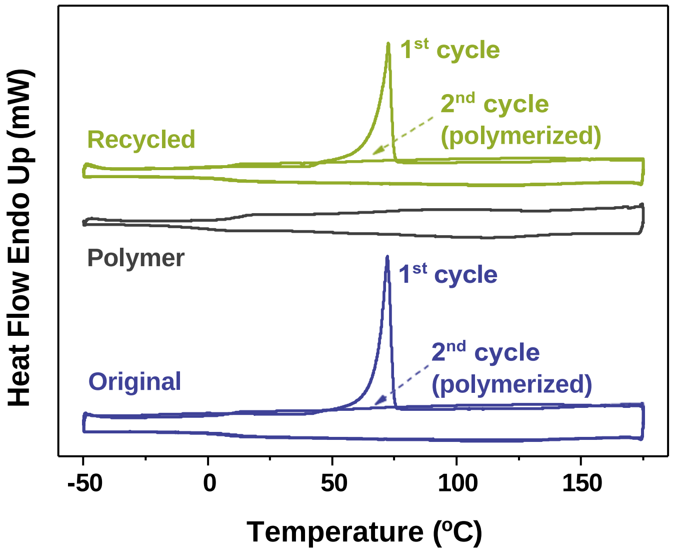

Figure S15. DSC thermograms for the original TAH monomer, poly(TAH), and chemical recycled TAH monomer obtained from poly(TAH) by heating from -50 °C to 175 °C with a temperature-increasing rate of 5 °C/min.

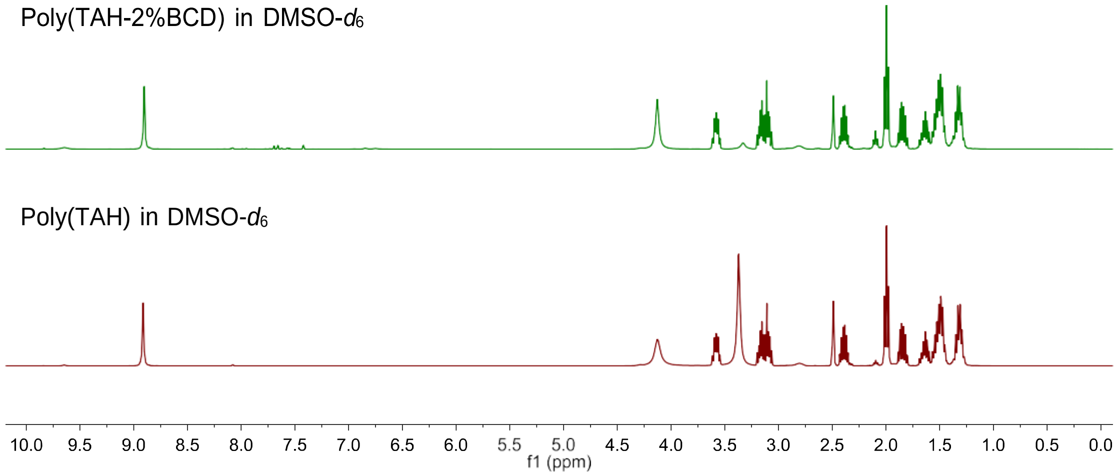

Figure S16. ^{1}H NMR spectra of poly(TAH) and poly(TAH-2% BCD) (DMSO-d_6, 400 MHz, 298 K).

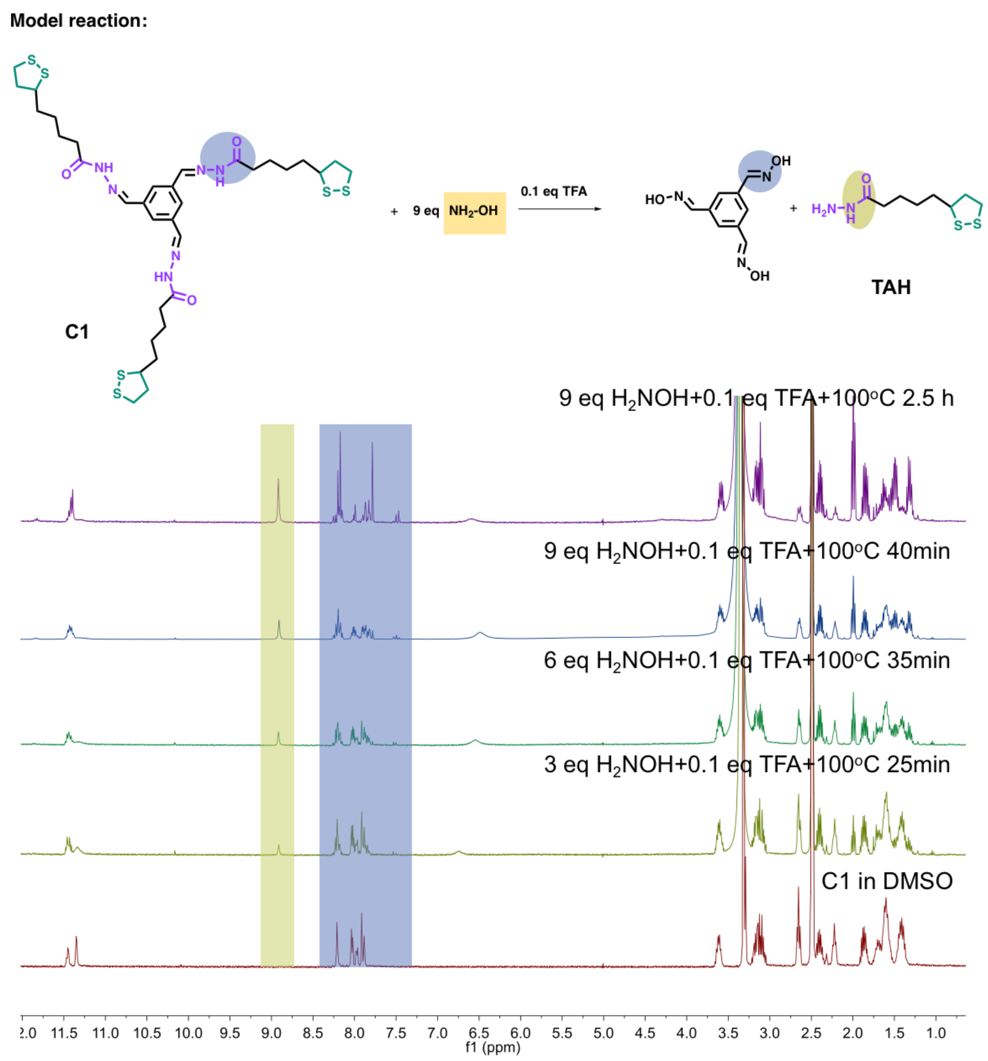

Figure S17. ^{1}H NMR spectra for model reaction with external hydroxylamine and catalytic amount of TFA (DMSO-d_6, 400 MHz).

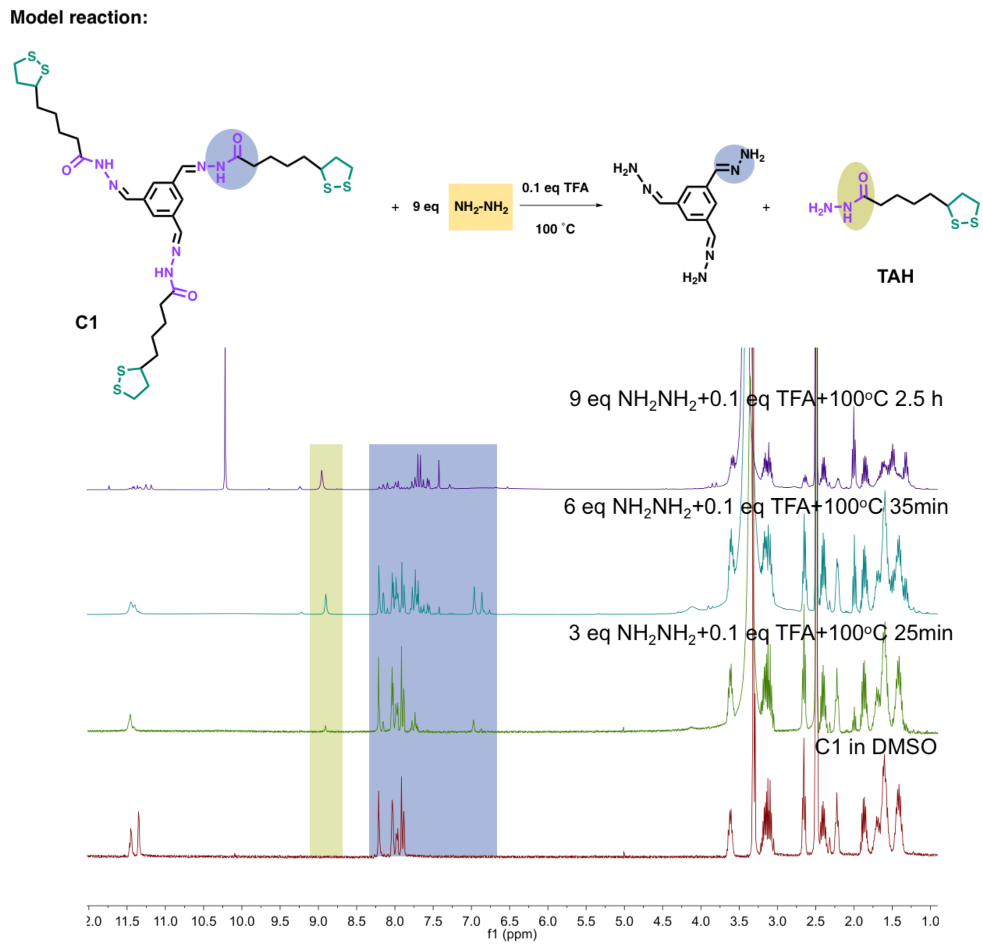

Figure S18. ^{1}H NMR spectra for model reaction with external hydrazine and catalytic amount of TFA (DMSO-d_6, 400 MHz).

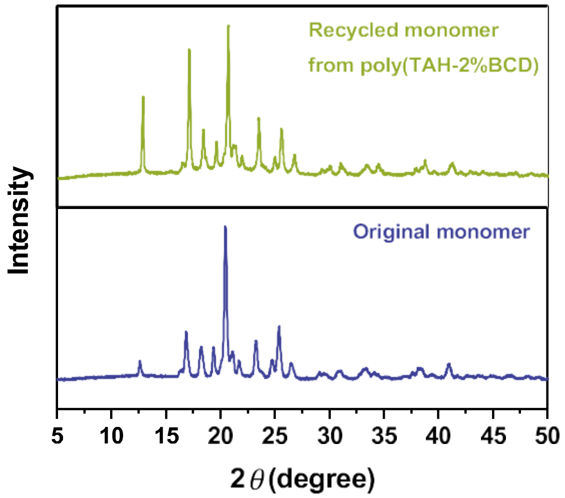

Figure S19. XRD data of the original TAH monomers and chemical recycled TAH monomers from poly(TAH-2% BCD).

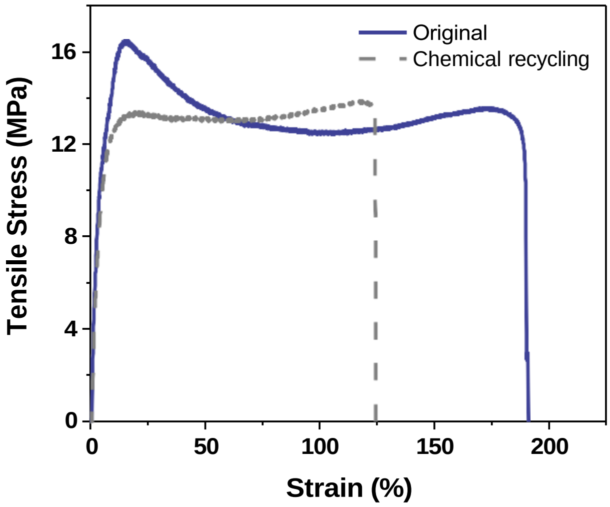

Figure S20. Stress-strain curves of chemical recycled poly(TAH-2%BCD).

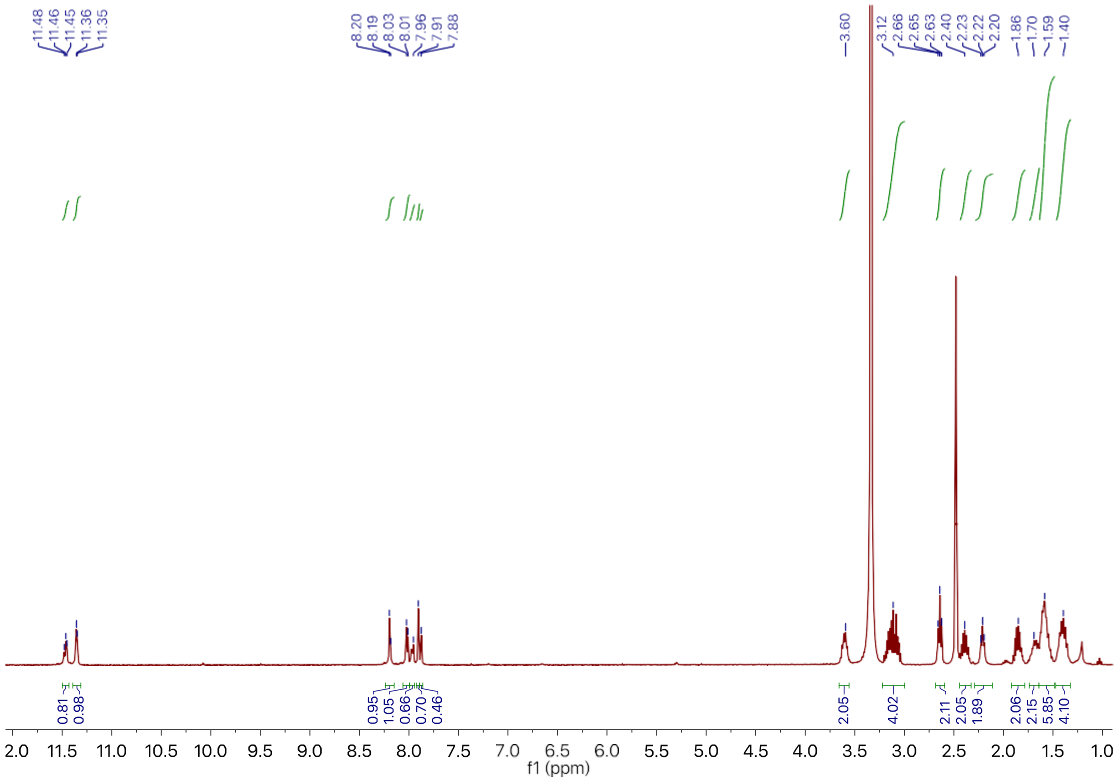

Figure S21. ^{1}H NMR Spectra of Compound **C1**(DMSO-d_6, 400 MHz, 298 K). The asterisked signals at δ 2.49 and 3.33 (^{1}H) are due to partially non-deuterated residues of DMSO-d_6 and water.

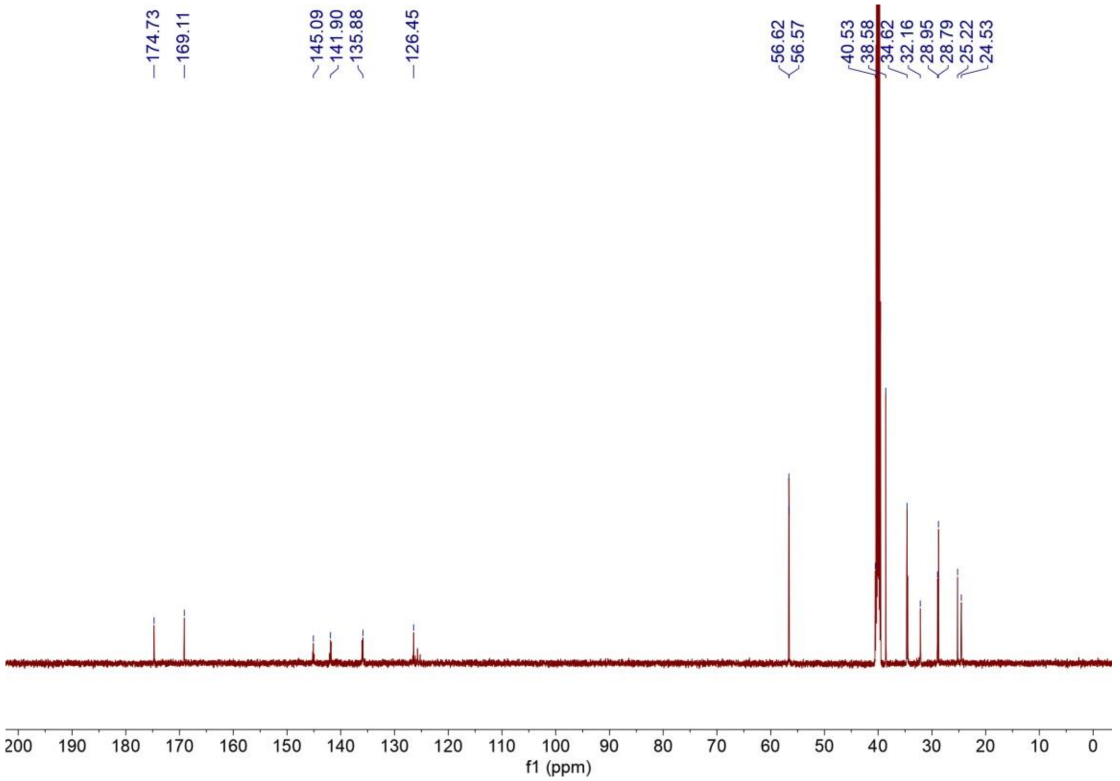

Figure S22. ^{13}C NMR Spectra of Compound **C1** (DMSO-d_6, 151 MHz, 298 K).

HRMS Spectra of Compound C1

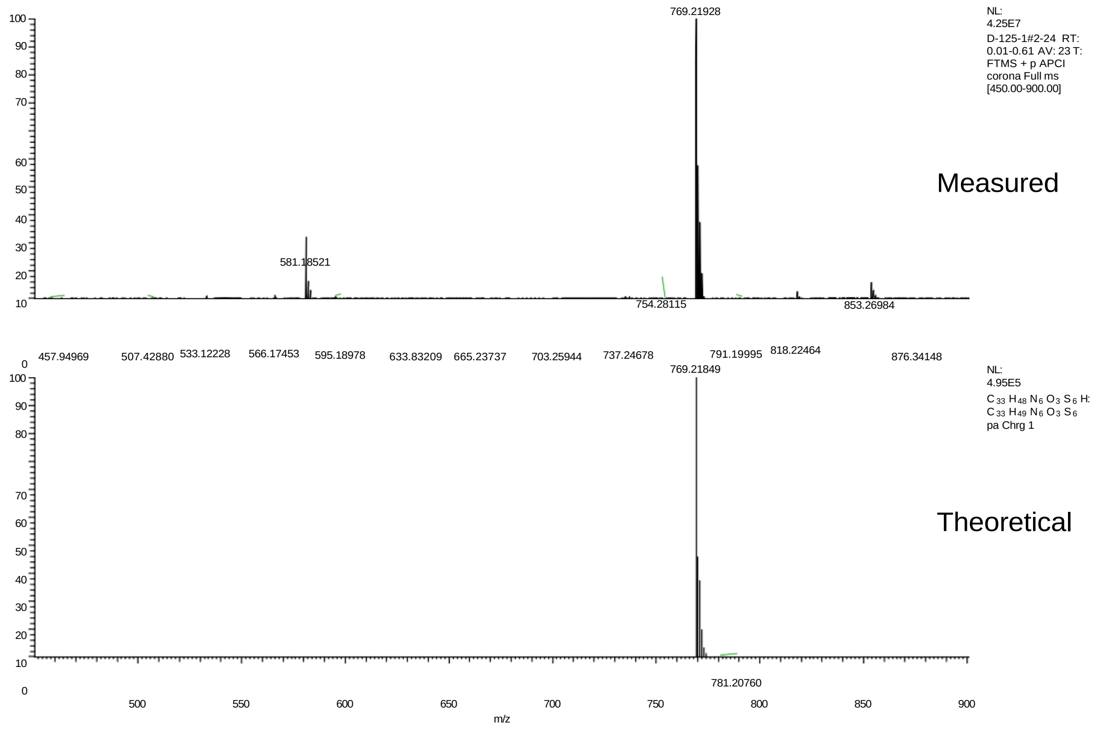

Figure S23. HRMS spectra of **C1**, LTQ Orbitrap XL (APCI+).

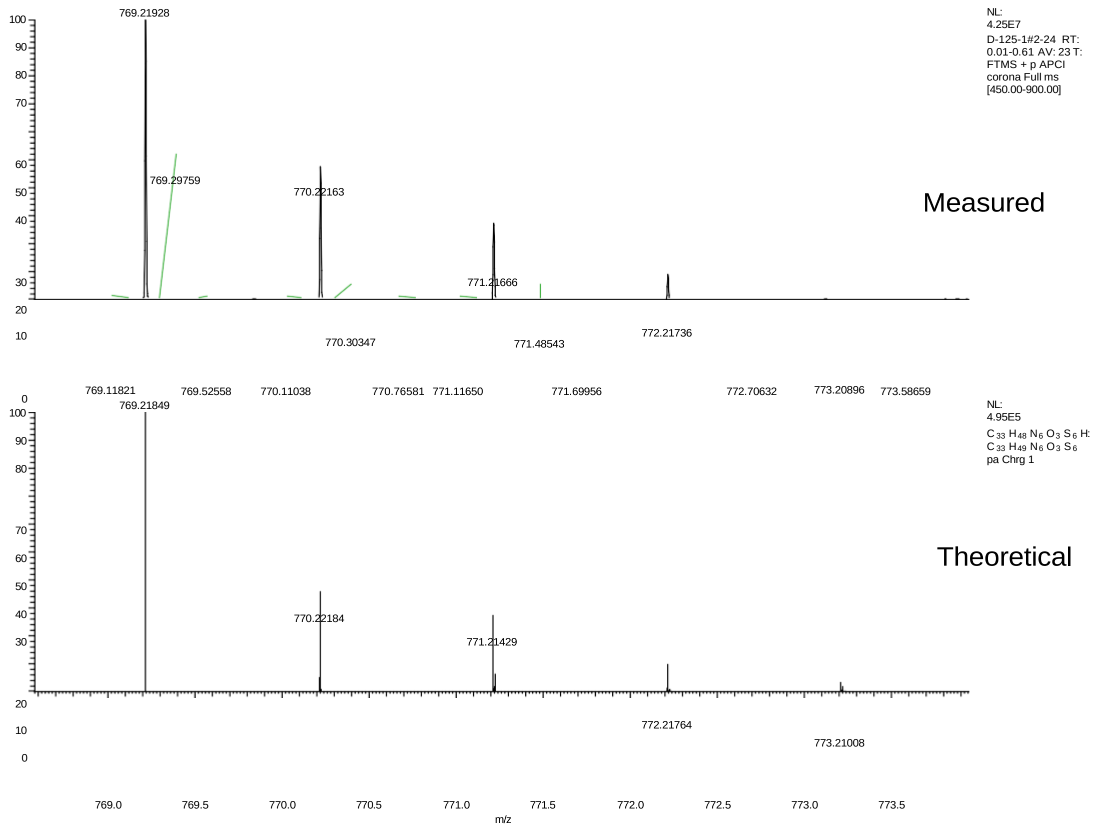

Figure S24. HRMS spectra (zoomed in) of **C1**, LTQ Orbitrap XL (APCI+).

6. Reference

[1] Y. Deng, Q. Zhang, C. Shi, R. Toyoda, D. H. Qu, H. Tian, B. L. Feringa, *Sci. Adv.* **2022**, *8*, abk3286.

[2] M. Röttger, T. Domenech, R. Van Der Weegen, A. Breuillac, R. Nicola,ÿL. Leibler, *Science*, **2017**, *356*, 62–65.
